# Credit assignment to state-independent task representations and its relationship with model-based decision making

**DOI:** 10.1073/pnas.1821647116

**Published:** 2019-07-18

**Authors:** Nitzan Shahar, Rani Moran, Tobias U. Hauser, Rogier A. Kievit, Daniel McNamee, Michael Moutoussis, NSPN Consortium, Raymond J. Dolan

**Affiliations:** ^a^Wellcome Centre for Human Neuroimaging, University College London, WC1N 3BG London, United Kingdom;; ^b^Department for Imaging Neurosciences, Max Planck University College London Centre for Computational Psychiatry and Ageing Research, WC1B 5EH London, United Kingdom;; ^c^Medical Research Council (MRC) Cognition and Brain Sciences Unit, University of Cambridge, CB2 7EF Cambridge, United Kingdom

**Keywords:** reinforcement learning, decision making, motor learning

## Abstract

Model-free learning enables an agent to make better decisions based on prior experience while representing only minimal knowledge about an environment’s structure. It is generally assumed that model-free state representations are based on outcome-relevant features of the environment. Here, we challenge this assumption by providing evidence that a putative model-free system assigns credit to task representations that are irrelevant to an outcome. We examined data from 769 individuals performing a well-described 2-step reward decision task where stimulus identity but not spatial-motor aspects of the task predicted reward. We show that participants assigned value to spatial-motor representations despite it being outcome irrelevant. Strikingly, spatial-motor value associations affected behavior across all outcome-relevant features and stages of the task, consistent with credit assignment to low-level state-independent task representations. Individual difference analyses suggested that the impact of spatial-motor value formation was attenuated for individuals who showed greater deployment of goal-directed (model-based) strategies. Our findings highlight a need for a reconsideration of how model-free representations are formed and regulated according to the structure of the environment.

## Significance

It is widely accepted that agents learn action values based on experience in a “model-free” manner (i.e., without holding a model of the environment). Environments usually embody many features, where a subset is considered relevant for model-free outcome learning. In this study, we show that a putative model-free system assigns credit to outcome-irrelevant task representations, regardless of stimulus features. The degree of impact of these associations is strongly linked to deployment of model-based strategies. Our findings motivate a reconsideration of how model-free representations are formed and regulated according to the structure of the environment.

In learning from outcomes, a decision maker can exploit at least 2 distinct neurocognitive systems: a model-free system and a more sophisticated model-based system (1). While model-based learning strategies incorporate explicit knowledge about the structure of the environment (2), model-free learning predicts outcomes based solely on the success of previously taken actions (3). To enable accurate action–outcome predictions, both systems are faced with a challenge, namely that only a portion of the information in the environment is predictive of a desired outcome. This entails that both the model-based and model-free systems should base their learning on action–outcome-relevant information alone.

Consider a child who has had a few samples of a new food. The spatial position of food on the table and whether the right or left hand was used to bring food to the child’s mouth are usually considered irrelevant to the impact of its consumption. Learning should reflect knowledge that the food carries the same value even if placed at different spatial positions on the table or if a different effector is required to obtain it. Disregarding value associations for spatial-motor aspects can be challenging when, as often is the case in the real world, these aspects are task relevant and are actively held in mind during task performance (4, 5). Such situations pose a challenge of maintaining a strict separation between active and accessible task-relevant action representations (e.g., currently, the food can only be grasped with a certain movement) and representations that support action value learning (e.g., learning the value of the food, disregarding the movement).

While the model-based system can be instructed that certain task aspects do not predict an outcome, a fundamental question arises as to whether and how a model-free system “knows” what to ignore given that it has only minimal knowledge regarding environmental structure. Previous studies examining model-based/model-free contributions to choice (2, 6–10) consider outcome-relevant model-free representations alone, implicitly assuming that outcome-irrelevant information does not impact learning. For example, a model-free system might evaluate different objects (e.g., images) with regard to their prediction of an outcome based solely on their identity and disregard spatial position and the motor action required to select them. However, the possibility that model-free learning is affected by outcome-irrelevant, yet task-relevant, information has not been previously explored.

Here, we examine the extent to which outcome-irrelevant model-free associations influence decision making. We administered a widely used 2-stage decision task (2, 6–12) to 769 adolescents. The task required subjects to navigate in a 2-stage transition maze to gain rewards, and this allowed us to disentangle a contribution of model-based and model-free systems to decision making. Importantly, reward probability depended on stimulus identity (images) but not on spatial-motor information (left/right locations and keys). Both sequential analysis and computational modeling suggested a credit assignment to spatial-motor task aspects in a state-independent manner (that is, regardless of task states, stages, or stimulus identity). Individual differences analysis suggested that a greater deployment of model-based strategies at the first stage was related to a lesser influence of spatial-motor model-free associations on decision making. These findings suggest a need for revision in our understanding of model-free representations and how these are regulated according to the structure of the task.

## Results

**Fig. 1.**
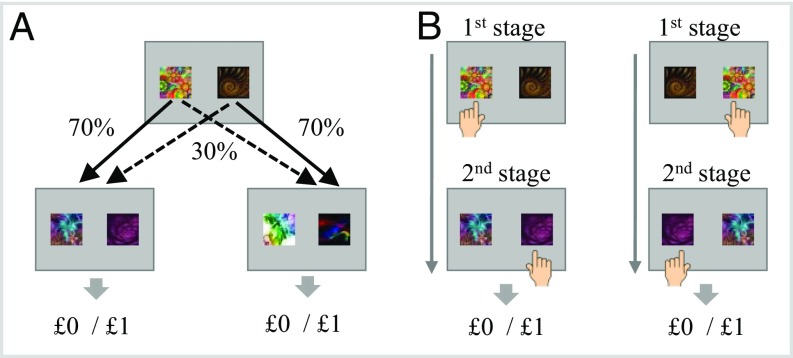
Schematic of the 2-step task. (*A*) At a first stage, participants choose between 2 options (represented by abstract fractal images) that determine the presentation of 1 of 2 second-stage states according to a fixed transition probability of 70% (“common”) or 30% (“rare”). At a second stage, participants also choose between 2 fractals to gain a reward (£0 or £1 play pounds). (*B*) Fractals were randomly assigned on each trial and stage to the right/left side of the screen. Participants indicated their choice by pressing a corresponding left/right arrow key. Therefore, the same fractal could be selected by either a left or right key press, and a fractal’s excepted value was unrelated to location on screen or the motor effector response used to report a choice. The panel illustrates 2 random trial sequences where a common transition took place. These trial sequences demonstrate that the same fractal selection could have been made with relation to different motor effector responses. Additional task information can be found in *SI Appendix*.

We studied a large sample of healthy volunteers (ages 14 to 24 y) who had completed a 2-step decision task (2) at 3 distinct time points repeated after ∼6 and 18 mo after the first measurement (*n* = 769, 63, and 568 for the 3 time measurements) (13). In the task, participants were asked to navigate between 2 stages to gain rewards (Fig. 1). The second stage included 2 pairs of stimuli (i.e., fractal images). To reach these, participants made first-stage choices that probabilistically determine the fractals presented at the second stage. Fractals were randomly allocated at each stage and each trial to the right/left side of the screen.

Participants were instructed to use a corresponding right/left key press to indicate their fractal selection. Therefore, the location of the fractal on the screen and the response used to indicate the selection were ostensibly task relevant. However, at each stage and each trial, fractal identity alone, but not spatial-motor aspects, predicted expected reward (Fig. 1). This allowed us to disentangle credit assignment to fractal as opposed to spatial-motor representations.

### Consecutive Trial Analysis.

We start by evaluating the impact of spatial-motor model-free (i.e., MF_spatial-motor_) associations on participants’ behavior using model-agnostic measures. Across analyses, we examined whether reward history affected the probability of response key selection on the next trial (*n* + 1). Note that, in this task, response key and fractal location are perfectly confounded. For simplicity, we address value associations only for the right/left response key (rather than fractal location).

#### Within-state analysis.

**Fig. 2.**
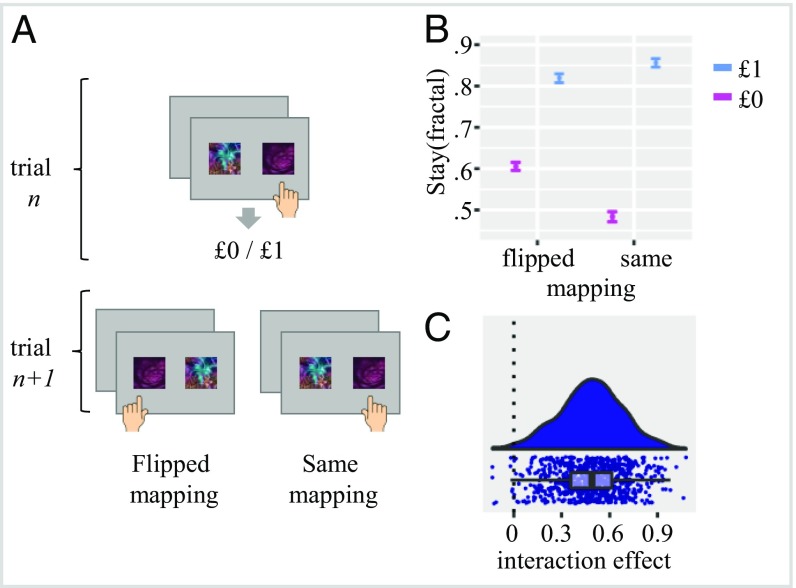
Within-state effects of reward on fractal and response key selection. (*A*) Example for a within-state trial sequence, where the same pair of second-stage fractals was offered with either the same or a flipped response mapping. (*B*) Effect of outcome (rewarded vs. unrewarded) and mapping (flipped vs. same) on the probability of choosing the same fractal at trial *n* + 1. Results highlight a tendency to repeat fractal selection after a rewarded trial. Notably, a greater effect of reward was evident when the fractal was mapped to the same compared with the alternative response key. This indicates that the effects of reward are evident at the level of the relevant fractal but also at the level of the outcome-irrelevant response key. Error bars represent 95% confidence intervals. (*C*) Raincloud plot (34) showing individual scores for the outcome × mapping interaction effect (as calculated in a mixed effect regression). Positive values indicate greater involvement of spatial-motor value associations on choice behavior.

We examined participants’ choices when making second-stage decisions as a function of outcome (unrewarded vs. rewarded) and response mapping (repeated or flipped fractal-to-key mapping) exclusively for trials where the same second-stage state repeated itself (Fig. 2*A*). We assumed that, for a chooser with no influence of MF_spatial-motor_ associations on behavior, the effect of reward will be exactly the same regardless of mapping. However, if there is some involvement of MF_spatial-motor_ associations, we would expect an outcome × mapping interaction such that a greater effect of reward on fractal selection would be evident when a response mapping is repeated vs. flipped. As an example, consider a participant who selected Fractal A with a “right” response key and obtained a reward (Fig. 2*A*). Since the predicted value of Fractal A increased, on the next trial the participant should be more likely to pick Fractal A again. However, if reinforcement value was also assigned to the right response key, then there should be an enhanced tendency to press that same key after a reward (compared with no reward). Consequently, the latter influence will act to increase or decrease the relative tendency to choose a specific fractal conditional on mapping repetition (i.e., reward × mapping interaction).

To examine this hypothesis, we calculated a mixed effect logistic regression (14) where outcome (unrewarded vs. rewarded), mapping (flipped vs. same), and their paired interaction were entered as fixed and random effects predicting the probability that the participant will repeat the same fractal selection (i.e., fractal stay probability). We found a statistically significant outcome × mapping interaction effect [χ^2^_(1)_ = 392.09, *P* < 0.001], showing a larger effect of reward on fractal stay probability when the chosen fractal was associated with the same key on both the *n* and *n* + 1 trials. Specifically, when mapping was flipped, reward increased fractal stay probability by 21.39% on average compared with 37.26% when fractal response mapping was repeated (Fig. 2*B*).

This result demonstrates that an effect of reward was dependent on the fractal effector response mapping, despite the fact that fractal alone predicted reward in this task. One possibility is that value was assigned to a combination of fractal and response key (for example, a different value was assigned for Fractal A with right response key and Fractal A with left response key). However, a second possibility is that credit was assigned to some extent to the response key independent of the state and outcome-relevant features of the stimuli. To arbitrate between these possibilities, we now examine trials where a different pair of fractals was offered at trial *n* compared with *n* + 1.

#### Between-states analysis.

**Fig. 3.**
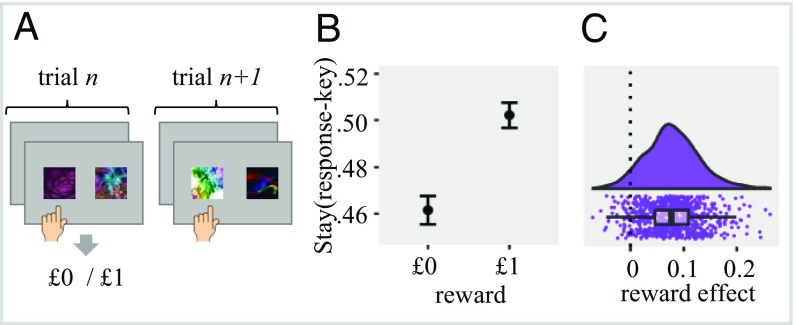
Between-state effect of reward on response key selection. (*A*) Example for sequence of trials included in the analysis, where a different pair of fractals was offered in the current and next second-stage state. (*B*) Effect of previous reward (rewarded vs. unrewarded) on the chance that the participant will select the same response key as in trial *n* + 1. The result reflects that value was assigned to the response key independent of task states or fractal identity. Error bars represents 95% confidence intervals. (*C*) Raincloud plot (34) showing individual scores for the outcome effect (as calculated in a mixed effect regression). Positive values indicate greater involvement of spatial-motor value associations on choice behavior.

To explore whether MF_spatial-motor_ associations affected performance across state and stimulus features, we examined participants’ choices at the second stage in trials where the pair of fractal presented at the trial *n* differed from the one presented in the following *n* + 1 trial (Fig. 3*A*). We calculated a mixed effect logistic regression with outcome (unrewarded vs. rewarded) predicting the probability that participants will repeat their right/left response key selection (i.e., response key stay probability). We found a statistically significant outcome effect [χ^2^_(1)_ = 101.1, *P* < 0.001], showing that participants were 4.07% more likely to repeat the response key selection at trial *n* + 1 when it was rewarded vs. unrewarded at trial *n* (Fig. 3*B*).

The between-state sequential effect accords with within-state effects reported above, suggesting that participants assigned value to spatial-motor information. While the within-state analysis could reflect a value assignment to the response key dependent on the state and/or related fractal, this explanation is inadequate for the between-state effect. The between-state analysis suggests that, even when a different pair of fractals was offered, reward had an effect on the next trial response key selection. We believe that this can only be explained under an assumption that value was assigned to the response key also, independent of the outcome-relevant fractals. Still, value assignment might be stage dependent so that individuals assigned value to response keys for the specific task stage, thus reflecting some dependency on outcome-relevant features of the task. To examine whether value assignment to response keys is independent of all outcome-relevant features (stages, states, and fractal identity), we examined next behavior at the first stage as a function of choice and reward at the previous second stage.

#### Between-stages effects.

**Fig. 4.**
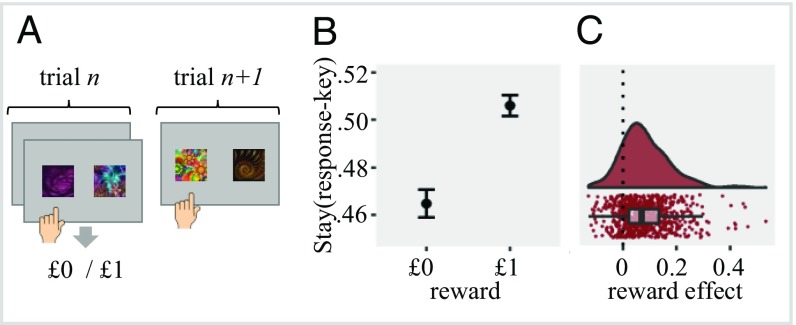
Between-stage effect of reward on response key selection. (*A*) Example for sequence of trials included in the analysis where we examine response key selection at the first stage of trial *n* + 1 as a function of response key selection at the second stage and reward at trial *n*. (*B*) Effect of reward (rewarded vs. unrewarded) on the chance that the participant will select in the first stage of trial *n* + 1 the same response key selected at the second stage of trial *n*. Results suggest that value was assigned to the outcome-irrelevant response key (or fractal location) independent of task stage, state, or fractal identity. Error bars represents 95% confidence intervals. (*C*) Raincloud plot (34) showing individual scores for the outcome effect (as calculated in the mixed effect regression). Positive values indicate greater involvement of spatial-motor value associations on choice behavior.

We examined whether MF_spatial-motor_ representations affected performance across task stages. Specifically, we calculated a mixed effect logistic regression with outcome (unrewarded vs. rewarded) predicting the probability that participants will repeat their response key selection at the second stage of trial *n* when performing a first-stage response key selection at trial *n* + 1 (Fig. 4*A*). We found a statistically significant outcome effect [χ^2^_(1)_ = 147.01, *P* < 0.001], showing that reward increased by 4.13% the probability that participants will select at the first stage of trial *n* + 1 the same response key selected at the second stage of trial *n* (Fig. 4*B*). This finding suggests that value was assigned to response keys and affected behavior independent of fractals, task states, and stages.

All 3 consecutive trial analyses (within state, between states and stages) did not correlate with age and were not reduced with practice (*SI Appendix*). Additional analysis demonstrated similar (but smaller) effects for the *n* + 2 trial (*SI Appendix*).

### Computational Modeling.

We next used computational modeling to examine and disentangle the relations between spatial-motor value associations and state representations for fractal learning (model-based and model free). We fitted 5 computational reinforcement learning models accounting for participants’ choices. 1) Model 1 (“null model”) did not include spatial-motor learning and accounts for participants choices by integrating fractal value assignment for both model-based and model-free systems (2). 2) Model 2 included the same fractal model-free and model-based learning as Model 1, with an additional separate learning system for MF_spatial-motor_. 3) Model 3 had the same fractal model-based learning as Model 1 but assumed that spatial-motor aspects affected model-free state representations and were weighted along with the fractal according to a linear function approximation to form state action values. 4) Model 4 had the same fractal model-based learning as Model 1 but also assumed that the model-free system held separate state action values for each pairing of fractal and response key. Finally, 5) Model 5 assumed that spatial-motor aspects affected both model-free and model-based state and transition matrix representations (model-agnostic analysis examining the influence of mapping on model-based decision making in the first stage can be found in *SI Appendix*). Therefore, in Model 5, both model-based and model-free systems held values for fractal response key pairing.

We used hierarchical fitting using expectation maximization with Laplace approximation method (15) and compared models by calculating Bayesian inference criteria (BIC), which penalizes the number of parameters (difference of 10 points or more is considered strong evidence, with lower scores indicating better fit) (16). We found a better fit for Model 2 vs. Model 1 (ΔBIC_int_ = 850.52), suggesting that the addition of an MF_spatial-motor_ influence improved the model’s ability to predict participants’ choices. For Model 3, 4, and 5, we found a worse fit compared with the null model (ΔBIC_int_ > 27,214.47).

To examine how well the best-fitting model (Model 2) predicted the observed behavior, we took the individual parameters from each participant and simulated data for 20 experiments with 1,000 trials each. We then estimated the 3 model-agnostic sequential scores from simulated data. Across individuals, we found estimates similar to those found with the empirical data, including the within-state effect (for mapping switches, reward increased fractal stay probability by 20.96% on average compared with 25.98% for mapping repetitions), between-states effect (reward increased response key stay probability by 2.55%), and between-stages effect (reward increased response key stay probability by 1.44%). At the individual level, we found good correspondence between model and observed data reflected in a statistically significant correlation between the 3 simulated and empirical effects (*r* = 0.41, CI_95%_ [95% confidence intervals] = 0.34 to 0.46, *P* < 0.001 for within-state effects; *r* = 0.41, CI_95%_ = 0.35 to 0.46 for between-state effects; *r* = 0.52, CI_95%_ = 0.25 to 0.38 for between-stage effects) (*SI Appendix*, Fig. S4). Therefore, both across and within individuals, we found a good fit between empirical and Model 2 simulated data. The same analysis for the next best-fitting model (Model 1) showed poor fit between the 3 model-agnostic scores from simulated compared with empirical data both across and between individuals (*SI Appendix*, Fig. S3).

The computational modeling results are, therefore, in line with the model-agnostic sequential analysis, suggesting that individuals cached values for spatial-motor representations independent of outcome-relevant task features. Furthermore, these results suggest that MF_spatial-motor_ was held and updated independent from outcome-relevant state representations.

### Relationship Between Spatial-Motor Model-Free Associations and Deployment of Model-Based Strategies.

The 2-step task makes it possible to estimate the extent to which participants act in accord with the transition structure of the task (i.e., reflecting model-based behavior). Specifically, by examining decisions at the first stage, one can estimate the extent to which individuals calculate a probability that their actions will lead to a certain second-stage state (2). Having a decision policy that exploits the probability that a certain first-stage action will lead to a desired second-stage state is considered a hallmark of model-based behavior (since the individual is deciding according to a model of the task transition structure). We conjectured that an individual’s task representation might include not only information regarding the transition structure but also, features of a current state that are relevant to an outcome. This builds on an assumption that MF_spatial-motor_ associations (assigning values to the motor response/spatial location) should be regulated according to a task mental representation. Consequently, we hypothesized that individuals with greater deployment of model-based strategies would also show less of an influence from MF_spatial-motor_ associations.

We specified 2 a priori latent factors capturing involvement of MF_spatial-motor_ associations and model-based processing on participants’ choices using structural equation modeling. Structural equation modeling is a multivariate method that combines factor analysis and multiple regression, allowing the estimation of structural relationships between latent constructs and their measured variables. Notably, latent factors are considered less noisy than their counterparts (17, 18), with structural equation modeling shown previously to increase reliability for 2-step task-related estimates (12). Here, we estimated the relationship between 2 a priori latent variables. 1) Model-free spatial-motor (MF_spatial-motor_) reflects a cognitive process of associating spatial-motor aspects of the task with reward outcomes. This we measured from 4 scores: a model parameter (Model 2) weighting the contribution of spatial-motor model-free associations to choice behavior (w_2_) (*Materials and Methods*) and the 3 sequential effects described above (within-state, between-state, and between-stage sequential effects) (Figs. 2–4). 2) model-based reflects an ability to incorporate knowledge about task transition structure when making a choice. This we measured from 3 scores: a model parameter (Model 2) weighting the contribution of model-based over model free to choice behavior (w_1_) (*Materials and Methods*) and 2 model-agnostic measures that were previously found to be tightly associated with model-based/model-free tradeoff, including the outcome (unrewarded vs. rewarded) × transition (uncommon vs. common) interaction effect on choice at *n* + 1 (first stage) (2, 12) and the transition effect on reaction time at the second stage (7, 8, 12) (*SI Appendix*).

We fitted a confirmatory factor model and found an acceptable model fit χ^2^_(4)_ = 69.16, *P* < 0.001; root mean square error of approximation = 0.074 (0.057 to 0.092); comparative fit index = 0.913; standardized root mean square residual = 0.041. Inspection of the covariance among the 2 latent factors showed a negative correlation between MF_spatial-motor_ and model-based learning (standardized covariance = −0.32, *P* < 0.001), suggesting that high–model-based participants also exhibited lower influence of MF_spatial-motor_ associations on decision making (*SI Appendix*, Fig. S5).

## Discussion

Natural environments are feature rich, and only a subset of these features is considered to predict action–outcome associations. An agent’s ability to exploit relevant information to predict accurately an outcome is vital for adaptive, goal-directed behavior (19, 20). While previous studies considered model-free credit assignment to outcome-relevant features of a task (2, 6–9), here we asked how, and to what extent, a model-free system avoids credit assignment to outcome-irrelevant task representations. Notably, both model-agnostic and computational analyses suggest that participants assigned value to outcome-irrelevant spatial-motor aspects of the task. We highlight here what seems to be an obligatory model-free system credit assignment to outcome-irrelevant task representations.

Our finding raises a question as to the nature of the observed spatial-motor model-free associations with respect to the stimulus. For example, after having reached for a grape placed on the left side of the table with a right hand gesture, could value be assigned to the hand gesture and/or location of the grape regardless of its visual features? We found evidence that credit is assigned to spatial-motor representations across task states and stages and in a stimulus-independent manner. This suggests that a model-free system assigns credit to low-level task representations, regardless of an assignment to outcome-relevant features of the task. This conclusion is in line with diverse findings from animal studies (21–23), focal brain damage (24, 25), and human learning studies (26, 27), which seem to show that stimulus and action-related value encoding are realized within distinct neural networks.

How can a model-free system that bears only minimal knowledge regarding environment structure know what representations should be susceptible or immune to credit assignment (28, 29). Insights arise from the observed negative relationship that we found between the deployment of model-based strategies and the impact of spatial-motor model-free representations. This association suggests that those who decide according to knowledge of a state transition structure (including future states in their decisions) are also those who display a lesser influence from low-level spatial-motor value associations. Thus, we speculate that participants’ mental map of the environment might be more elaborated then previously described (2, 6–9) and include information about which features of the environment best predict a reward outcome. This information might then guide top-down regulatory processes that dampen the influence of outcome-irrelevant model-free associations.

Stimulus-independent motor learning might have particular relevance for interpretation of studies in patient populations. For example, compulsive behavior (10, 30), substance use (31), and obesity (32) are linked to less goal-directed model-based influences. It is nevertheless the case that these conditions are also strongly associated with specific motor actions (e.g., eating, checking, etc.). Therefore, a tendency to form motor–outcome associations might be a form of learning manifest in the context of a relevant specific outcome (e.g., movements related to food or safety). We suggest that future studies might ask whether putative value-related impairments are in fact more domain specific than previously thought.

Finally, our findings also correspond with current assumptions regarding how human cognition assembles and regulates states (1, 29). The reinforcement learning literature defines a “state” as a collection of information that is relevant to a given decision (20). Our findings might entail a tendency for certain representations to be considered part of a state, thus affecting decisions in a state-intrinsic manner. We speculate that the learning system has a requirement to segregate task representations (assumed to be actively held in working memory) into state-extrinsic/intrinsic information according to outcome relevancy. When working memory resources are low (or more demanding), an individual’s ability to keep an accurate intrinsic- vs. extrinsic-state differentiation between task representation might be attenuated, leading to higher involvement of outcome-irrelevant task representations on value-based choices.

There are limitations to our study as well as additional questions arising from our findings. First, our design could not determine whether the effect of spatial-motor reward associations is due to unregulated learning (i.e., regulating the formation of irrelevant associations), unregulated decision (i.e., regulating the influence of these associations on behavior), or both. Second, our manipulation of spatial location and motor response is perfectly confounded, with additional studies needed to disentangle the contribution of those domains. Finally, above the stimulus-independent spatial-motor associations, participants might also assign credit for a combination of stimulus and motor response/object location.

In summary, we uncover a substantial effect of model-free spatial-motor outcome-irrelevant learning on behavior. Our computational modeling suggests that cached value for the spatial-motor aspects is generalized across distinct state features. This highlights the need to address how states are structured and represented within the model-free system.

## Materials and Methods

### Participants.

Written informed consent was given for all participants. The Cambridge Central Research Ethics Committee approved the study (12/EE/0250). Data were obtained from a community-based longitudinal sample of young volunteers (ages 14 to 24 y) living in the United Kingdom [Neuroscience in Psychiatry Network’s study (13, 33)]. Our final dataset after exclusions (*SI Appendix*) included 769 individuals at Time I (371 males, 398 females, mean age = 19.06, range = 14.10 to 24.99), 63 at Time II (34 males, 29 females, mean age = 19.41, range = 14.93 to 24.90), and 568 at Time III (284 males, 284 females, mean age = 20.30, range = 15.11 to 26.48). Additional information can be found in *SI Appendix*.

### Procedure.

At all 3 time measurements, participants were invited to a laboratory session in one of the United Kingdom’s collaborating institutions (13). The mean time gap between Times I and II was 6.48 mo (range = 5.04 to 8.04 mo), and between Times I and III, it was 17.78 mo (range = 11.76 to 31.44 mo). At each measurement session, participants completed computer-based cognitive evaluations and clinical assessments. At the end of the assessment day, participants were paid a fixed amount plus a bonus based on performance.

### Reinforcement Learning Models.

We fit a reinforcement learning model to participants’ behavior, where we estimated the predicted value of each choice at each trial based on reward history. We used a temporal difference learning algorithm, wherein predicted values for each choice (i.e., Q value) are updated according to a prediction error teaching signal. We tested 5 models, each differing with respect to how spatial-motor elements are integrated to affect decision making.

#### Model 1 (null model).

Here, we integrated 2 value components following Daw et al. (2): 1) model-free fractal value—reflecting the amount of previous reward that followed this fractal selection—and 2) model-based fractal value—reflecting the highest value of the 2 fractals reached by a common/rare transition after a first-stage action. Let f_1_/f_2_ be the fractals selected at the first/second stage of the task, and let reward at trial *n* be r_(n)_ε{0,1}. Fractal model-free Q values were initialized to 0 at the beginning of the experiment and updated at the end of each trial according to a state–action–reward–state–action reward prediction error algorithm (20) for first-stage choices,

$${{\text{Q}}^{\text{Fractal}}}_{\left(\text{f1},n+\text{1}\right)}{{=\text{Q}}^{\text{Fractal}}}_{\left(\text{f1},n\right)}{+\alpha }_{1}\left({{\text{Q}}^{\text{Fractal}}}_{\left(\text{f2},n\right)}-{{\text{Q}}^{\text{Fractal}}}_{\left(\text{f1},n\right)}\right){+\alpha }_{1}\lambda_{1}\left({\text{r}}_{\left(\text{n}\right)}-{{\text{Q}}^{\text{Fractal}}}_{\left(\text{f2},n\right)}\right),$$ [1]

and second-stage choices,

$${{\text{Q}}^{\text{Fractal}}}_{\left(\text{f2},n+\text{1}\right)}{{=\text{Q}}^{\text{Fractal}}}_{\left(\text{f2},n\right)}{+\alpha }_{1}\left({\text{r}}_{\left(\text{n}\right)}-{{\text{Q}}^{\text{Fractal}}}_{\left(\text{f2},n\right)}\right),$$ [2]

where α_1_ was a fractal learning rate (free parameter) and λ_1_ was an eligibility trace (free parameter) capturing the effect of the second-stage prediction error on first-stage fractal value. Next, the model-based (MB) learning strategy incorporated the empirical transition probabilities and second-stage Q^Fractal^ values to estimate the value of first-stage actions for each Fractal F according to

$${{\text{Q}}^{\text{MB}}}_{\left(\text{F},n\right)}=\text{P}\left({\text{s}}_{2}|\text{F}\right)\times \text{max}\left({{\text{Q}}^{\text{Fractal}}}_{\left(\text{s2},n\right)}\right)\;+\text{P}\left({\text{s}}_{3}|\text{F}\right)\times \text{max}\left({{\text{Q}}^{\text{Fractal}}}_{\left(\text{s3},n\right)}\right),$$ [3]

where s_2_ and s_3_ represented the 2 states in the second stage (Fig. 1) and P(s_2_|F) and P(s_3_|F) represented the transition probability. We then calculated an integrated Q value for each fractal F, with a *w* parameter-quantified model-based vs. model-free tradeoff in first-stage actions:

$${{\text{Q}}^{\text{net}}}_{\left(\text{F,n}\right)}{=\text{w}}_{1}{{\cdot \text{Q}}^{\text{MB}}}_{\left(\text{F,n}\right)}+\left(1-{\text{w}}_{1}\right)\cdot {{\text{Q}}^{\text{Fractal}}}_{\left(\text{F,n}\right)}.$$ [4]

Note that model-based and model-free predictions are identical at the second stage; therefore, at the second stage, the integration of model-based and model free leads to

$${{\text{Q}}^{\text{net}}}_{\left(\text{F,n}\right)}={{\text{Q}}^{\text{Fractal}}}_{\left(\text{F,n}\right)}.$$ [5]

We further examined an extension of Model 1, where we included 2 different learning rates for mapping repetitions vs. switches (assuming that fractal value assignment might be noisier when mapping flips). However, this did not improve the model fit (*SI Appendix*).

#### Model 2.

Model 2 assumed that fractal learning (model-based and model free) was the same as Model 1. However, here we integrated a separate system that assigned model-free values to response key (separate learning rate and prediction error). Thus, for Model 2, we also updated response key model-free values at the end of each trial. Let k1 and k2 be the response keys for stages 1 and 2, respectively. Response key model-free values were updated for the first stage according to

$${{\text{Q}}^{\text{Key}}}_{\left(\text{k1,n}+\text{1}\right)}{{=\text{Q}}^{\text{Key}}}_{\left(\text{k1,n}\right)}{+\alpha }_{2}\lambda_{2}\left({\text{r}}_{\left(\text{n}\right)}-{{\text{Q}}^{\text{Key}}}_{\left(\text{k1,n}\right)}\right)$$ [6]

and for the second stage according to

$${{\text{Q}}^{\text{Key}}}_{\left(\text{k2,n}+\text{1}\right)}={{\text{Q}}^{\text{Key}}}_{\left(\text{k2,n}\right)}{+\alpha }_{2}\left(1-\lambda_{2}\right)\left({\text{r}}_{\left(\text{n}\right)}-{{\text{Q}}^{\text{Key}}}_{\left(\text{k2,n}\right)}\right),$$ [7]

where α_2_ was a response key learning rate (free parameter) and λ_2_ was a free parameter allowing differentiation between credit assignment to the first and second actions when they differ. We then calculated Q^net^ for the first stage according to

$${{\text{Q}}^{\text{net}}}_{\left(\text{F,n}\right)}=w_{1}\cdot {{\text{Q}}^{\text{MB}}}_{\left(\text{F,n}\right)}+\left(1-w_{1}\right){{\cdot \text{Q}}^{\text{Fractal}}}_{\left(\text{F,n}\right)}+w_{2}\cdot {{\text{Q}}^{\text{Key}}}_{\left(\text{K,n}\right)},$$ [8]

where K is the response key for selecting fractal F, and for the second stage according to

$${{\text{Q}}^{\text{net}}}_{\left(\text{F,n}\right)}={{\text{Q}}^{\text{Fractal}}}_{\left(\text{F,n}\right)}+w_{2}{{\cdot \text{Q}}^{\text{Key}}}_{\left(\text{K,n}\right)}.$$ [9]

We further examined an extension of Model 2 where we included 2 different learning rates (α_2_) for mapping repetitions vs. switches. However, this did not improve the fit of this model (*SI Appendix*).

#### Model 3.

The model included the same model-based fractal learning as Model 1 (Eqs. **2** and **3** were used to estimate second- and first-stage model-based values, respectively). However, model-free state action values (i.e., Q^MF^) were estimated using a linear approximation function, where fractals and response keys served as weighted features. Specifically, Q values for the model-free system were calculated according to

$${{\text{Q}}^{\text{MF}}}_{\left(\text{s,a}\right)}={\text{w}}_{1}{\text{f}}_{1\left(\text{s,a}\right)}{+\ldots +\text{w}}_{\text{n}}{\text{f}}_{\text{n}\left(\text{s,a}\right)},$$ [10]

where s and a denote specific state and action, respectively. f_i(s,a)_ε{0,1} represents whether a feature was available (1) or not (0) for a certain state and action. f_1_ to f_6_ represented the 6 fractals in the task, and f_7_ and f_8_ represented the 2 response keys. w_1_ to w_8_ were the respective weights, which were updated at the end of each trial. First-stage weights were updated according to

$${\text{w}}_{\text{i}\left(\text{n}+\text{1}\right)}{=\text{w}}_{\text{i}\left(\text{n}\right)}{+\alpha }_{2}\cdot \left({{\text{Q}}^{\mathrm{MF}}}_{\left(\text{s2,a2}\right)}-{{\text{Q}}^{\mathrm{MF}}}_{\left(\text{s1,a1}\right)}\right)\cdot {\text{f}}_{\text{i}\left(\text{s1,a1}\right)}{+\alpha }_{2}{\cdot \lambda }_{1}\cdot \left({\text{r}}_{\left(\text{n}\right)}-{{\text{Q}}^{\text{MF}}}_{\left(\text{s2,a2}\right)}\right)\cdot {\text{f}}_{\text{i}\left(\text{s1,a1}\right)},$$ [11]

and second-stage weights were updated according to

$${\text{w}}_{\text{i}\left(\text{n}+\text{1}\right)}{=\text{w}}_{\text{i}\left(\text{n}\right)}{+\alpha }_{2}\cdot \left({\text{r}}_{\left(\text{n}\right)}-{{\text{Q}}^{\text{MF}}}_{\left(\text{s2,a2}\right)}\right)\cdot {\text{f}}_{\text{i}\left(\text{s2,a2}\right)}.$$ [12]

Finally, Q^net^ for first stage was calculated according to

$${{\text{Q}}^{\text{net}}}_{\left(\text{F,n}\right)}={\text{w}}^{\mathrm{MB}}\cdot {{\text{Q}}^{\text{MB}}}_{\left(\text{F,n}\right)}+\left(1-{\text{w}}^{\mathrm{MB}}\right){{\cdot \text{Q}}^{\text{MF}}}_{\left(\text{s,a,n}\right)}.$$ [13]

Since in this model, Q^MB^ and Q^MF^ were no longer identical for the second stage (model-free values here are also driven by the response key mapping, which is ignored by the model-based system), we also weighted the second-stage Q^net^ according to Eq. **13**. We further examined an extension of Model 3, where we included 4 response key features (right/left for first/second stages). However, this did not improve the fit of this model (*SI Appendix*).

#### Model 4.

The model included the same model-based fractal learning as Model 1 (Eqs. **2** and **3**). However, in contrast to Model 1, we assumed that model-free state action values are held separately for each fractal response key combination. Therefore, in this model, we update 12 Q^MF^ values for each combination of fractal and response keys. For first stage, Q^MF^ values were updated according to

$${{\text{Q}}^{\text{MF}}}_{\left(\text{f1,k1,n}+\text{1}\right)}{{=\text{Q}}^{\text{MF}}}_{\left(\text{f1,k1,n}\right)}+\alpha_{2}\left({{\text{Q}}^{\text{MF}}}_{\left(\text{f2,k2,n}\right)}-{{\text{Q}}^{\text{MF}}}_{\left(\text{f1,k1,n}\right)}\right){+\alpha }_{2}\lambda_{1}\left({\text{r}}_{\left(\text{n}\right)}-{{\text{Q}}^{\text{MF}}}_{\left(\text{f2,k2,n}\right)}\right),$$ [14]

and for second stage, they were updated according to

$${{\text{Q}}^{\text{MF}}}_{\left(\text{f2,k2,n}+\text{1}\right)}={{\text{Q}}^{\text{MF}}}_{\left(\text{f2,k2,n}\right)}{+\alpha }_{2}\left({\text{r}}_{\left(\text{n}\right)}-{{\text{Q}}^{\text{MF}}}_{\left(\text{f2,k2,n}\right)}\right).$$ [15]

Finally, Q^net^ for first and second stage was calculated according to

$${{\text{Q}}^{\text{net}}}_{\left(\text{n}\right)}{=\text{w}}_{1}\cdot {{\text{Q}}^{\text{MB}}}_{\left(\text{F,n}\right)}+\left(1-{\text{w}}_{1}\right)\cdot {{\text{Q}}^{\text{MF}}}_{\left(\text{F,K,n}\right)}.$$ [16]

Therefore, in this model, the pairing of fractal F and response key K is conjunctively defined with the stimulus. Note that, in this model, Q^MB^ and Q^MF^ were no longer identical for the second stage, and therefore, we also weighted the second-stage Q^net^ according to Eq. **16**.

#### Model 5.

We assumed that both the model-based and model-free systems held different state representation for each pair of fractals with each available mapping. We, therefore, updated Q^MF^ similar to Model 4 (Eqs. **14** and **15**). However, here the model-based system also calculated the probability of reaching each pair of fractals in the second stage with each available mapping. We define 4 second-stage states for each pair of fractals and response mapping (s_2A_, s_2b_, s_3A_, s_3B_). Q values for the model-based system in the first stage were calculated as follows:

$${{\text{Q}}^{\text{MB}}}_{\left(\text{F,n}\right)}=\text{P}\left({\text{s}}_{2\text{A}}|\text{F}\right)\times \text{max}\left({{\text{Q}}^{\text{Fractal}}}_{\left(\text{s2A,n}\right)}\right)+\text{P}\left({\text{s}}_{2\text{B}}|\text{F}\right)\times \text{max}\left({{\text{Q}}^{\text{Fractal}}}_{\left(\text{s2B,n}\right)}\right)+\text{P}\left({\text{s}}_{3\text{A}}|\text{F}\right)\times \text{max}\left({{\text{Q}}^{\text{Fractal}}}_{\left(\text{s3A,n}\right)}\right)+\text{P}\left({\text{s}}_{3\text{B}}|\text{F}\right)\times \text{max}\left({{\text{Q}}^{\text{Fractal}}}_{\left(\text{s3B,n}\right)}\right),$$ [17]

where probabilities for common transition were considered to be 35% and probabilities for uncommon transition were considered to be 15% following the current task design. Since model-based and model-free values are again identical in this model in the second stage, we used Eq. **16** to integrate model-free and model-based Q values only for the first stage.

Equally for all of the models, we added a choice bias value accounting for 1) fractal perseveration [tendency to repeat fractal selection regardless of reward for first stage only following previous studies (2)], 2) response key perseveration (tendency to repeat response key selection regardless of reward), and 3) response key bias (reflecting a tendency to use one response key more than the other due, for example, to hand dominancy effects). *SI Appendix* has additional information and models.

### Data and Code Availability.

Data and code can be found at https://osf.io/7dekj/?view_only=77bfbdb324db46d0aee5563759389aea.

## Supplementary Material

Supplementary File
