# Supplementary material for: Credit assignment to state-independent task representations and its relationship with model-based decision making
Source: Proc Natl Acad Sci U S A. 2019 Jul 18;116(32):15871–6. doi: 10.1073/pnas.1821647116 (PMC6689934; doi:10.1073/pnas.1821647116)
Supplement: Supplementary File [file pnas.1821647116.sapp.pdf]

PNAS
www.pnas.org

Supplementary Information for:

Credit-assignment to state-independent task representations and its relationship with model-based decision-making

Nitzan Shahar[1,2], Rani Moran[1,2], Tobias U. Hauser[1,2], Rogier A. Kievit[2,3], Daniel McNamee[1,2], Michael Moutoussis[1,2], NSPN consortium, Raymond J. Dolan[1,2]

[1] Wellcome Centre for Human Neuroimaging, University College London, London WC1N 3BG, UK

[2] Max Planck University College London Centre for Computational Psychiatry and Ageing Research, London WC1B 5EH, UK

[3]MRC Cognition and Brain Sciences Unit, University of Cambridge, 15 Chaucer Rd, Cambridge CB2 7EF

[*] Correspondence to: Nitzan Shahar
Wellcome Centre for Human Neuroimaging
University College London
12 Queen Square, London WC1N 3BG, UK
Tel: +44 (0)20 3448 4362
Email: shahar.nitzan@gmail.com

This PDF file includes:

- Supplementary text
- Figs. S1 to S9
- Tables S1 to S3
- References for SI reference citations

www.pnas.org/cgi/doi/10.1073/pnas.1821647116

Consecutive trial analysis

Mixed-effect regression. For the sequential analyses, data was collapsed across the three time-points. Choice was predicted using 'lmer' R package (1) logistic regression (with a Laplace approximation and bound optimization by quadratic approximation). P-values were obtained from likelihood ratio tests of the full model with the fixed effect in question against a model without that fixed effect (the same random effect was kept in both models).

Sequential effects on trial *n*+2. To explore whether spatial-motor value associations influenced choice beyond the *n* + 1 trial, we repeated the same sequential analyses reported in the main text, only predicting choice at the *n*+2 trial (without controlling for states and choices at the *n*+1 trial):

(1) Within-state analysis. We analyzed second-stage choices where the same pair of fractals was offered in the *n* and *n+2* trials. We found an effect for outcome x response-mapping interaction on choice ($\chi^2_{(1)}$=73.99, p<.001), with a similar direction on fractal stay probability as in the *n*+1 analysis. Specifically, when fractal-mapping was switched, reward increased fractal stay probability by 22% on average compared with 25.90% when fractal-response mapping was repeated (see Figure S1).

(2) Between-states analysis. We analyzed second-stage choices where a different pair of fractals was offered in the *n* and *n*+2 trials. We found a significant effect for reward *n* ($\chi^2_{(1)}$=4.47 , p<.05) such that participants were 0.74% more likely to repeat the motor responses at trial *n* + 2 after it was rewarded at trial (see Figure S1).

(3) Between-stages analysis. We analyzed first-stage choices at the *n*+2 trial as a function of reward and response-key selection at the second-stage of trial *n*. We found a statistically significant outcome effect ($\chi^2_{(1)}$=6.81 ,p<.01), showing that reward increased by 0.56% the probability that participants will select at the first-stage of trial *n* + 2 the same response-key selected at the second-stage of trial *n* (see Figure S1).

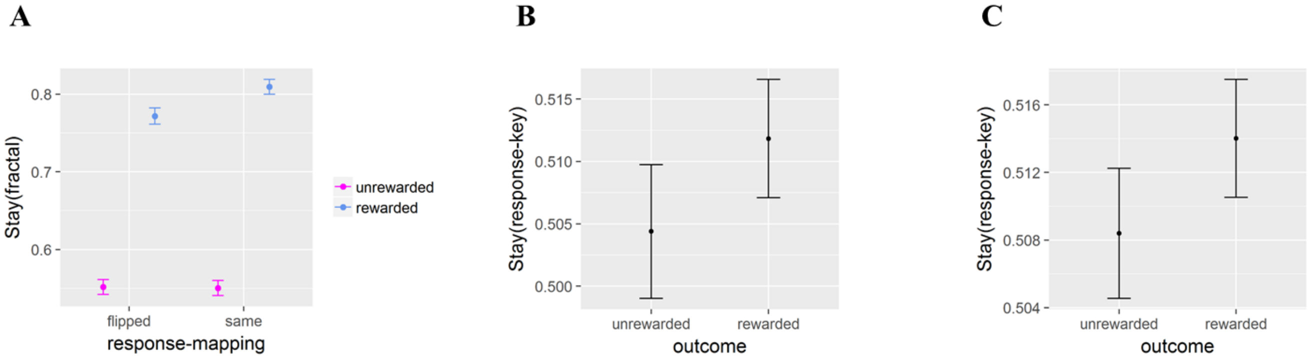

Figure S1. Effects on the *n*+2 trial **(A)** within-state effect of previous outcome and fractal-mapping repetition on fractal stay probability, **(B)** between-states effect of previous reward on response-key selection for second-stage choice where a different pair of fractals was offered on both trials, and **(C)** between-stages effect showing the effect of reward on the

probability that participants will perform at the first stage of the n+2 trial the same response-key that was selected at the second-stage on the *n* trial.

Correlations with age. We were interested to explore whether age correlated with a tendency to assign value to spatial-motor aspects of the task. We estimated Pearson correlation for age and three model-agnostic scores for spatial-motor value assignment, only for the first time measurements, where we had participants that spanned the entire age range 14.1 to 25, (mean age=19.06). We found no evidence for a correlation of age with either a within-state (r=.01, p=.80), between-states (r=-.01, p=.91) or between-stages effects (r=-.07, p=.06).

Practice effects on spatial-motor value associations. Here, we examine whether the effect of spatial-motor value association on behavior was reduced with practice (e.g., due to the fact that participants learned from experience that spatial-motor aspects of the task are non-informative). To do this we re-calculated the three model-agnostic scores quantifying the influence of spatial-motor value associations on behavior (within-state, between-states and between-stages effects, see main text). The three scores were calculated separately for each task-part (first vs. second half of the task) and session (Time I/II/III) (see Figure S2). Descriptive results suggested no systematic reduction in the spatial-motor scores with practice (see Figure S2). We examined an effect of task-part (start vs. end) separately on each of the three scores (across time measurements). We found no effect for within-state (Figure S2A; $t_{(781)}$=-.59, p=.55) or between-states scores (Figure S2B; $t_{(781)}$=-.1.33, p=.18). The between-stages estimates showed a slightly lower spatial-motor value effect for the first (3.32%) compared to the second (4.83%) half of the task (Figure S2C; $t_{(781)}$=-2.98, p<.01). Thus, there was no evidence for a reduction in spatial-motor value associations during task performance.

We next examined whether a slight increase in spatial-motor model-free estimates was accompanied by a corresponding reduction in model-based estimates. We repeated the same analysis with model-based estimates (indicated by the interaction effect of previous transition and reward over the stay probability in the first-stage) and again found no evidence for a change between the first and second part of the task (Figure S2D; $t_{(781)}$=-.1.16, p=.24).

Thus, we found no evidence supporting the idea that spatial-motor model-free associations reduce during practice. We speculate that a slight increase in one of the spatial-motor model-free scores is possibly a consequence of reduced cognitive control in the second part of the task (e.g., due to fatigue). Here, reduced cognitive control resources could lead to attenuated regulation over low-level spatial-motor value association, which increase the influence of low-level spatial-motor model-free associations. However, since the increase in spatial-motor model-free estimates was not observed consistently for all estimates, and was not accompanied with a corresponding reduction in model-based estimates, we believe further studies involving a direct manipulation of cognitive control resource availability are needed to investigate the cause of this effect as well as whether it is specific to the between-stages estimate.

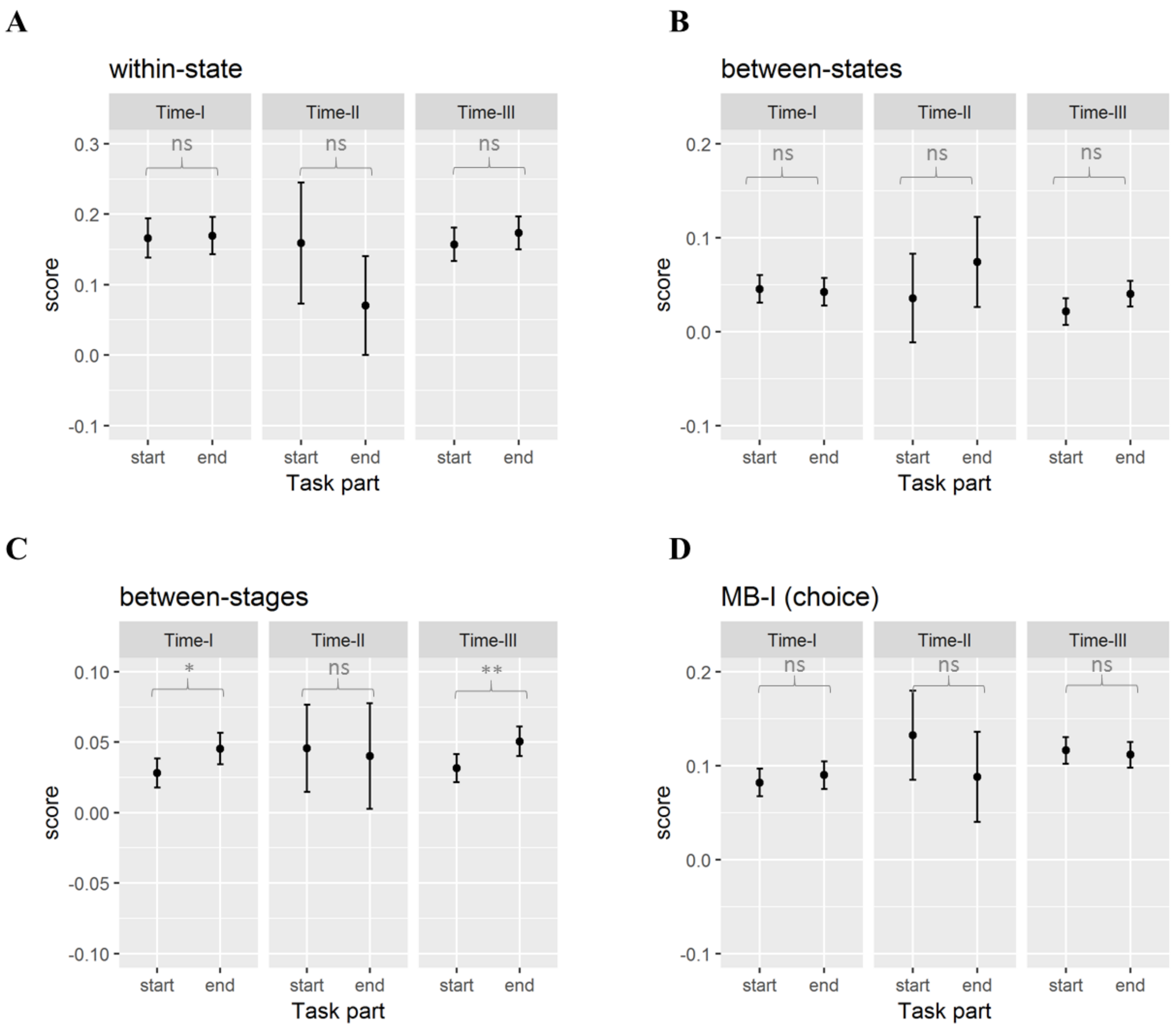

Figure S2. Spatial-motor model-free and model-based estimates (y-axis, see Consecutive trial section in main text for estimates description) across task-part (x-axis, start vs. end) and time measurement (Time-I/II/II, N=769, 63 and 568, respectively). (**A**) Effect on within-state spatial-motor value estimates, (**B**) Between-states spatial-motor value estimates, and (**C**) Between-stages spatial-motor value estimates (see main text). (**D**) Model-based scores estimated by the interaction of previous transition and reward over the tendency to stick with first-stage choice. Overall, these results provide no evidence for a reduction in spatial-motor value associations either across time or with practice. Error-bars represents 95% CI. * p<.05, **p<.01.

Computational Modeling

Calculating choice probability. Equally for all the models, after calculating an integrated Q-value (i.e., Qnet, as described in the main text for each model), we added a choice bias value which was an integrated value for three components: (a) fractal perseveration (tendency to repeat fractal selection regardless of reward, for first-stage only following previous studies (2)), (b) response-key perseveration (tendency to repeat response-key selection regardless of reward), and (c) response-key bias (reflecting a tendency to use one response-key more than the other, due for example to hand dominancy effects):

$$bias_{(a,n)} = p_1 \cdot \mathrm{Stay}^{\mathrm{Fractal}}_{(n)} + p_2 \cdot \mathrm{Stay}^{\mathrm{Response\text{-}key}}_{(n)} + p_3 \cdot \mathrm{Key}_{(n)}$$

$bias_{(a,n)}$ described the amount of bias for a certain action (a) at trial n. Here, $\mathrm{Stay}^{\mathrm{Fractal}} \epsilon \{0,1\}$ indicated whether the fractal was not selected (0) or selected (1) at the previous trial and p_1 was a free-parameter that could obtain either negative or positive values, allowing to account for a tendency to switch or repeat the previous fractal selection regardless of reward (following previous studies, p_1 was set to zero for second stage choices (2)). $\mathrm{Stay}^{\mathrm{Response\text{-}key}} \epsilon \{0,1\}$ indicated whether the current response-key was not selected (0) or selected (1) in the previous response. For first-stage choices, $\mathrm{Stay}^{\mathrm{Response\text{-}key}}$ was based on the response-key selected at the second-stage of the previous trial, and for the second-stage choices $\mathrm{Stay}^{\mathrm{Response\text{-}key}}$ was based on the response-key selected at the first-stage of the same trial. p_2 was a free-parameter that could obtain either negative or positive values, allowing to account for a tendency to switch or repeat the previous response-key selection regardless of reward. $\mathrm{Key}_{(n)} \epsilon \{0,1\}$ indicated a left (0) or right (1) response-key selection, and p_3 was a free-parameter that could obtain either negative or positive values, allowing to account for a general tendency to select the left or right response-key.

Finally, the probability of a first/second action was determined using a softmax, with a 1/β parameter representing the decision temperature:

$$\text{Eq. } P(a,t) = \frac{\exp(\beta[Q^{net}(a,n) + bias\,(a,n)])}{\sum_{a\prime} \exp(\beta[Q^{net}(a\prime,n) + bias(a\prime,n)])}$$

Model fitting. To obtain hierarchical fit we used expectation-maximisation with Laplace approximation method (3). In this approach, individual-participant parameters are treated as independent random effects sampled from Gaussian-population distributions (one distribution per parameter), whose means and variances are estimated. For model fitting we collapse all three time-points, searching for a single set of parameters that best predicted all available data (Note that Q-values were reset to zero at the first trial of each time-point measurement).

Additional models. Here we introduce slight variants to Model 1,2 and 3, where we aimed to examine whether a slightly different model performed better than the equivalent model described in the main text. None of these three supplementary models (Model 1A, 2A, 3A) outperformed the equivalent respective model reported in the main text (Model 1,2,3).

Model 1A: We extended Model 1 (where spatial-motor associations had no influence on behavior; Daw et al., 2011(2)) to include an assumption that credit assignment to fractals was less effective in states where the response-mapping switched vs. repeated compared to the

previous trial. For example, individuals could have remembered better which fractal they selected when the mapping did not switch, thus affecting credit assignment. To test this assumption we re-fitted Model 1, only this time we updated fractals' Q-values using one of two learning rates. For first-stage choices (see Eq 1, main text) we assigned α_1 if the response-mapping was repeated, and α_2 if it was switched compared with the previous trial. For second-stage choices (Eq 2, main text), we assigned α_1 if the same pair of fractals was offered with the same response-mapping as in the previous trial, or α_2 when the mapping was switched or a different pair of fractals was offered. We found a model with a single learning rate to have a better fit to the data compared with to a model with two learning rates (ΔBIC= 1115.1). Thus the former (Model 1) was used as our null model to allow comparison to other models.

Model 2A: We examined an extension to Model 2 (separate fractal and spatial-motor value learning), where we included two learning rates for the spatial-motor component, one for mapping repetitions and the other for mapping switches, allowing us to account for potential fluctuations in spatial-motor credit assignment due to mapping-repetitions. This model outperformed Model 1 where no spatial-motor learning took place (ΔBIC_{int}=705.31), but was worse than Model 2 which included spatial-motor learning with a single learning-rate (ΔBIC_{int}=145.19).

Model 3A: We elaborated Model 3 to include four response-key representations (two for each stage). Thus this Model had f1 to f6 representing the fractals and f7 to f10 representing response-keys for the first-stage (f7/8) and second-stage (f9/10). This model was worse than Model 3 which included two response-key features (ΔBIC_{int}=2352.57).

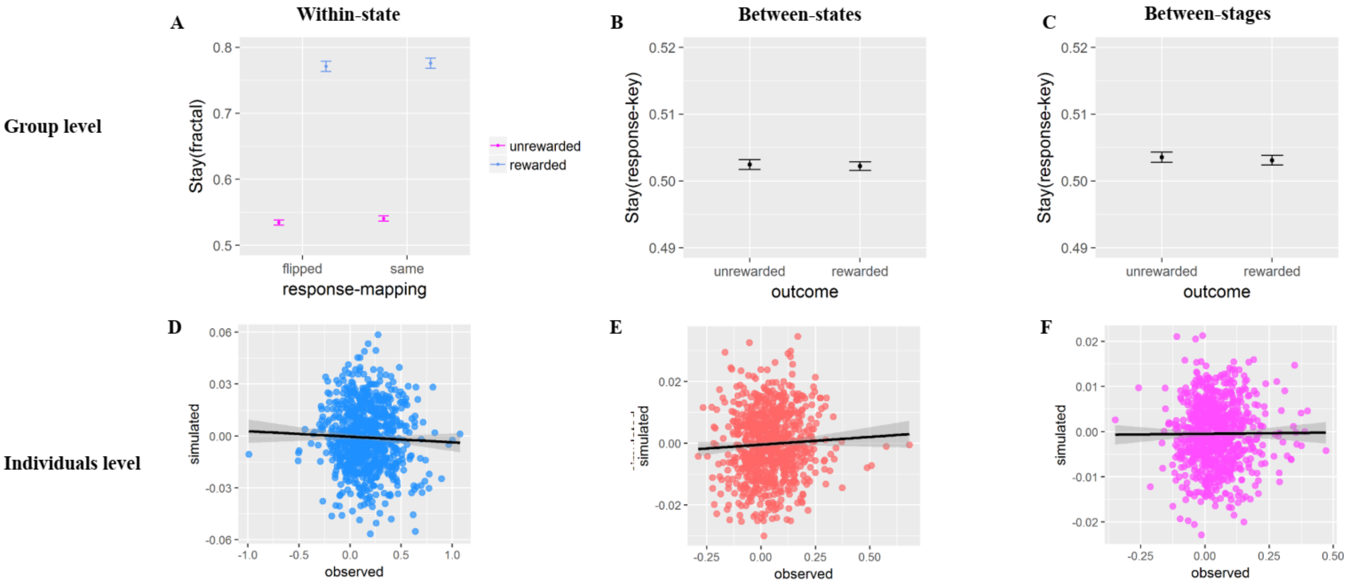

Figure S3. Model prediction and comparisons for Model 1. To obtain this data we simulated 1000 trials for 10 experiments per agent using the individual recovered parameters (Model 1).

(**A/B/C**) show the same within-state, between-states and between-stages effects described in the main text (see Figures 2-4), only calculated from simulated data. Specifically, for the within-state effect, we found that when fractal-mapping was switched, reward increased fractal stay probability by 23.61% on average compared with 23.52% when fractal-response mapping was repeated. For the between-states and between-stages effects we found that agents had ~0% difference on average in the probability to repeat the response-key selection at trial *n* + 1 when it was rewarded vs. unrewarded at trial *n*. **(D/E/F)** Correlations between the three sequential effects calculated from simulated and observed data for within-state scores (r=-.01, p=.33), between-states effects (r=.05,p=.11) and between-stages effects (r=.01,p=.80). Overall we found a poor fit between Model 1 predictions and the observed effects of spatial-motor value assignment both on the group level and at the individual level.

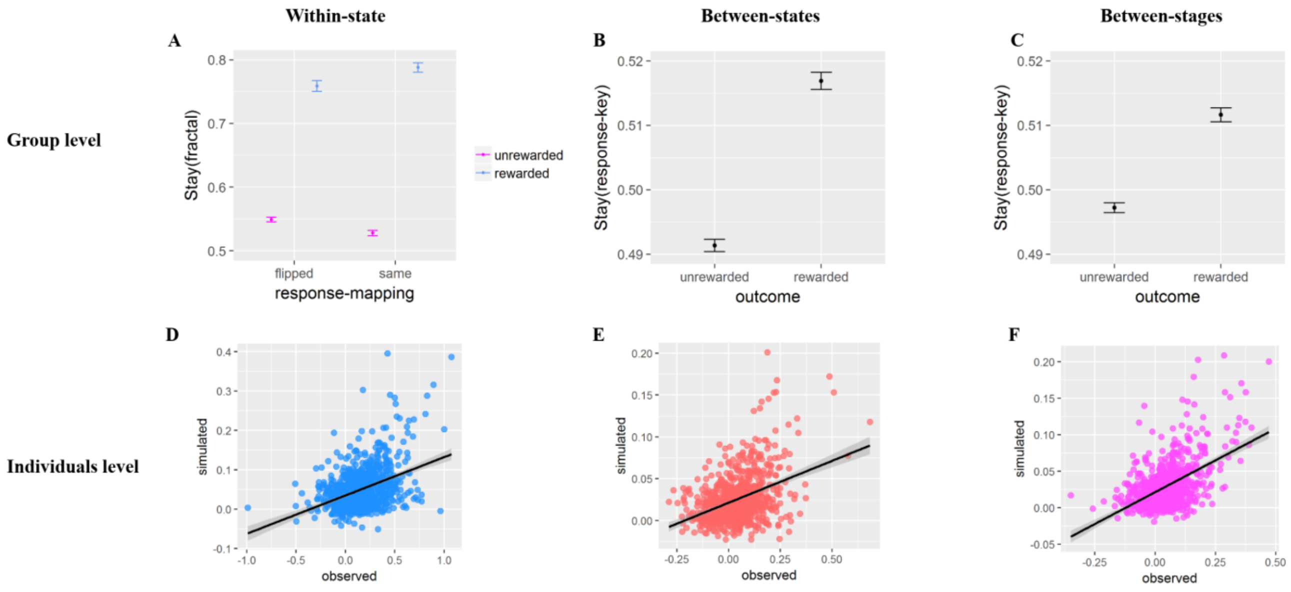

Figure S4. Model prediction and comparisons for Model 2. To obtain this data we simulated 1000 trials for 10 experiments per agent using the individual recovered parameters (Model 2). (**A/B/C**) show the same within-state, between-states and between-stages effects described in the main text (see Figures 2-4), only calculated from simulated data. Specifically, for the within-state effect, we found that when fractal-mapping was switched, reward increased fractal stay probability by 20.96% on average compared with 25.98% when fractal-response mapping was repeated. For the between-states and between-stages effects we found that agents had 2.55% and 1.44% difference on average, respectively, in the probability to repeat the response-key selection at trial *n* + 1 when it was rewarded vs. unrewarded at trial *n*. **(D/E/F)** Correlations between the three sequential effects calculated from simulated or observed data for within-state scores (r=.41, p<.001), between-states effects (r=.41,p<.001) and between-stage effects (r=.52,p<.001). Overall we found a very good fit between Model 2 prediction and observed spatial-motor effects so that both on the group level and at the

individual level Model 2 replicated the effects found for model-free spatial-motor associations.

Table S1. Descriptive statistics for Model 2 parameters.

**Parameter**	**mean**	**sd**	**Description**
α_1	0.33	0.23	Fractal learning rate
β	4.13	1.30	Inverse temperature
λ_1	0.56	0.19	Fractal eligibility trace
w_1	0.38	0.19	Model-based/free trade-off
p_1	0.19	0.12	Fractal perseveration
p_2	-0.02	0.04	Response-key perseveration
p_3	0.01	0.04	Response-key bias
w_2	0.23	0.09	Impact of spatial-motor model-free associations
α_2	0.29	0.22	Response-key learning rate
λ_2	0.35	0.11	first/second-stage response-key weighted credit assignment

Structural equation model (SEM)

Model-based indicators. The model-based (MB) latent factor predicted three variables: $w1$ parameter quantifying model-free/based trade-off (see above), and two model-agnostic measurements, previously shown to be tightly related with $w1$ model-parameter. This first model-agnostic score was interaction effects of transition (common vs. rare) and reward (rewarded vs. non-rewarded) in the previous trial on the probability of repeating a first stage choice at the next trial (2, 4). Unlike a model-free agent that is assumed to be affected by reward alone, and not transition, a model-based agent is assumed to make use of both reward and transition information. Specifically, model-based agent was assumed to increase the probability of repeating a first-stage choice only when the previous transition was common, while after uncommon transitions reward reduces the probability of repeating first-stage action (thus increasing the chance that the agent would return to the state where the reward was obtained). This translates into a positive interaction of previous reward and previous transition on the probability of repeat of the first-stage action for agents that are more model-based. The second model agnostic score was the reaction-time difference between common and uncommon transitions (5–7). This score was recently shown to be tightly related to the deployment of model-based strategy at the first-stage (7).

Model-free spatial-motor indicators. The model-free spatial-motor latent factor predicted four variables: *w2* parameter quantifying involvement of $MF_{spatial\text{-}motor,}$ and three model-agnostic scores described in the main text (a) *within-state effect* calculated as the interaction effect of outcome (rewarded vs. unrewarded) x response-mapping (repeated vs. flipped) on probability of repeating the fractal selection in the n+1 trial. (b) *between states effect* calculated as the effect of outcome (rewarded vs. unrewarded) on the probability the individual will repeat the same response-key selection at the n+1 trial, only for second-stage choices where the state was different between the two trials. (c) *between stages effect* calculated as the effect of outcome (rewarded vs. unrewarded) on the probability the individual will repeat the same response-key selection preformed at the second-stage of the n trials at the first-stage of n+1 trial.

Fitting. We fit a confirmatory factor model (CFA) using 'lavaan' package in R the Robust Maximum Likelihood estimator with a Yuan-Bentler scaled test statistic (MLR) to account for deviations from multivariate normality (8), and full information maximum likelihood estimation to account for missing data (under the assumption that data is missing at random). To evaluate model fit we rely on the Satorra-Bentler scaled test statistic along with the chi-squared test, the root mean square error of approximation (RMSEA) with its confidence interval, the comparative fit index (CFI), and the standardized root mean squared residuals (SRMR) (9). Using these indices, good fit was defined as: the value of the Satorra-Bentler scaling factor, RMSEA (acceptable fit<0.08, good fit <0.05), CFI (acceptable fit 0.95-0.97, good fit >0.97), and SRMR (acceptable fit 0.05-.10, good fit <0.05) for each model. Indicators beta coefficient are described in Table S2.

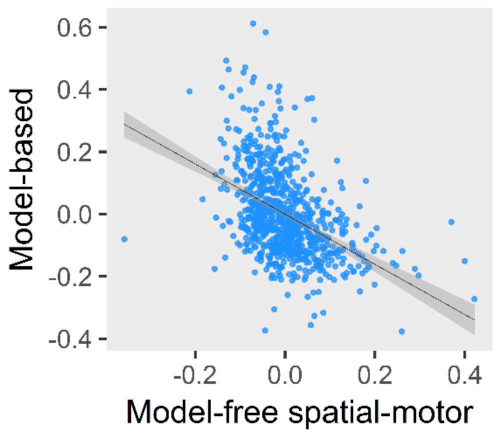

Figure S5. Scatter-plot demonstrating the relationship between a model-based and model-free spatial-motor latent factors as estimated using a confirmatory structural equation modeling (SEM). Results suggest that participants that had a stronger deployment of model-based strategies at the first-stage, also had lower impact of outcome-irrelevant task representations (spatial-motor) model-free learning.

Table S2. Standardized beta coefficient describing the relationship between latent variables and the observed indicators in the structural equation model

**Latent variable**	**Parameter**	**β**
Model-based	w_1	$.58^{***}$
	Model-agnostic score I (Reward x Transition on first-stage fractal choice repetition)	$.83^{***}$
	Model-agnostic score II (transition effect on reaction-times at the second-stage)	$.61^{***}$
Model-free spatial-motor	w_2	$.38^{***}$
	within-state effect	$.47^{***}$
	between-states effect	$.52^{***}$
	between-stages effect	$.40^{***}$

Note. ***p<.001

Method

Two-stage Decision Task. The task was the same as that developed by Daw at el., 2011, (2) and is described in Figure S6, and Figure 1 in main text. Participants were instructed to win as much reward (play pounds) as possible, and were told they would receive a payment bonus based on task performance. On each of the stages, subjects had to select one of two fractals. Each trial started by offering two first-stage fractals that appear at the bottom-left and bottom-right sides of the screen. Immediately after making a first-stage choice, the selected fractal appeared in a middle-upper position on the screen for 1.5 sec. The fractals offered during the second-stage were then added to the bottom-left and bottom-right sides of the screen, allowing participants to indicate their second-stage choice. Following the second-stage choice the second-stage selected fractal remained on the screen and a reward (gold coin), or no reward (red 'X' mark), appeared in center-bottom side of the screen for 1.5 seconds. The location of the fractals at each state (right vs. left) was randomly selected by the computer at each trial and stage. Participants were instructed to indicate their fractal choice by pressing a right/left arrow on a standard computer keyboard corresponding to the position where the chosen fractal was presented. Instructions noted that response-keys should be used to indicate fractal selection, and that fractals predict reward. Second-stage fractal predicted reward according to a random walk. We counterbalanced between participants two predefined random-walks with (probability range .2 to .8). Both the first and second-stage choice had a 2 seconds response deadline. If the 2 seconds deadline was exceeded 'Too late. No money earned.' was presented on screen for 1.5 seconds. Finally, each trial had an inter-trial interval that was randomly selected from a uniformed distribution ranging from 1 to 2 second. The task included 121 trials at Time-I & II and 201 trials at Time-III (a shorted version in Time I&II was given due to time constrained). A short break was provided after half of the trials. 50 practice trials where provided before the test phase. Practice and test phase included a different set of fractal images.

Instruction screen noted: "*Welcome to the 'Two step' Task! In this task you will have to make two decisions (at two steps) during each trial. At the first step you will choose between two pictures. Each of these will lead you to another pair of pictures. Each of the pictures of the second step can lead you to a reward (a play-pound), or to nothing (an 'X'). Some of the pictures in the second step USUALLY lead to reward, some only SOMETIMES. Use the LEFT ARROW and RIGHT ARROW keys to make your choices. Your task is to find out which is the best picture and get to it, in order to win points. Note, however, that 'the best' pictures will change every now and then, so you will have to keep checking! You will start with some practice rounds to get used to the task. The practice trials will not count towards your score. Feel free to ask the experimenter if anything is unclear at any point*"

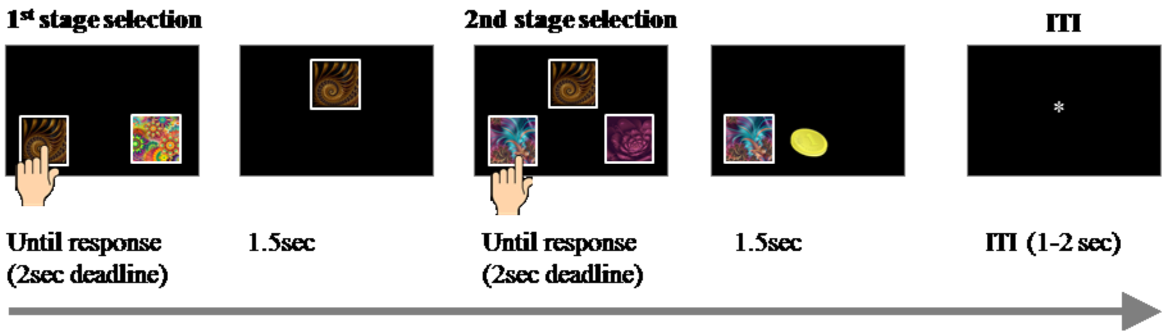

Figure S6. An example of trial sequence in the current two-step task.

Participants' recruitment. Participants were recruited by invitation via adverts in the community, local general practitioners (GP), schools and further education colleges. Recruitment was in an age-sex-stratified sample, for the following five age groups: 14-15, 16-17, 18-19, 20-21, and 22-24 years. Written informed consent was given for all participants (those aged 14-15 years gave written informed assent and their parents/legal guardian provided written informed consent). Participants were then invited to take part in a detailed in-lab behavioral assessments including computer-based evaluations (with the two-step task) and clinical assessments during three time points (sample size was N=783, 63, and 571 for the three time points, respectively). Our final dataset after exclusions (see below) included 769 individuals at Time-I (371 males, 398 females, mean age=19.06, range 14.10 to 24.99), 63 at Time-II (34 males, 29 females, mean age=19.41, range 14.93 to 24.90) and 568 at Time-III (284 males, 284 females, mean age=20.30, range 15.11 to 26.48). In this sample, 54 participants had data-sets for all three time measurements, 510 for two measurements (7 for Time I&II, 502 for Time I and III and 1 for Time II & III), and 220 with one measurement (206 Time I, 1 Time-II and 11 Time-III). Further details about recruitment, participants consent, and ethical approval can be found at Kiddle et al., (2018) (10).

Participant exclusion and pre-processing. We excluded participants that had more than 10% of the trial data missing due to technical malfunction (two at Time-I), did not respond on more than 10% of the trials (two at Time-I and one at Time-III), responded in the first-stage with the same response-key on more than 95% of the trials (three at Time-I), or had implausible RTs (below 150ms) on more than 20% of the trials (seven at Time-I) (4). This resulted in the inclusion of 769/63/568 individuals for Time-I to III, respectively. For the remaining two-stage task data, the first trial in each block, as well as trials with implausible RTs (below 150ms) were omitted from the analysis (1% of the overall trials).

Influence of model-free spatial-motor associations on monetary reward

In the current task, fractals were equally likely to be associated with right/left position/response-key each time they were offered for selection (i.e., random fractal to response-key mapping). To demonstrate the outcome-irrelevant nature of spatial-motor

aspects across the task, we simulated Model 2, using 1000 trials with 10 'experiments' per agent using individual recovered parameters. We found across agents a mean reward of 54.66%. To examine the influence of spatial-motor value associations alone on monetary gains we repeated the very same simulations, only this time 'shutting down' fractal-learning for both the model-based and model-free systems by fixing α_1 to zero (see Eq 1&2, main text). We found an average reward of 50.01%, demonstrating chance level monetary reward for spatial-motor value learning.

We repeated the same analysis with Model 2B where we introduced two learning-rates, one for mapping-repetitions and one for mapping-switches. Thus, this model is better able to account for stronger spatial-motor value learning when spatial-motor aspects might seem to predict the outcome, due to momentary co-presentations of fractals and response-keys. Simulation with individual recovery parameters resulted in above chance level 54.71% reward on average. Fixing α_1 to zero (no fractal learning for model-based and model-free systems) demonstrated again chance level monetary reward (50.00%).

These results demonstrate that spatial-motor value learning did not contribute to monetary rewards, thus showing the outcome-irrelevant nature of these associations.

Effect of mapping repetition on model-based estimates in the first-stage

We assumed that the mapping in the first-stage might influence the ability of participants to accurately follow the transition matrix. For example, it might be that when the response-mapping switched from one trial to the next, participants had a harder time to accurately select the fractal that would most likely lead the to the desired second-stage state. To examine this hypothesis, we examined whether the transition x outcome interaction effect on the probability of repeating first-stage choices (indexing model-based involvement; Daw et al., 2011(2)), is affected by mapping-repetitions compared to the previous trial. We found the three-way interaction of mapping (switched vs. repeated) x previous-outcome (unrewarded vs. rewarded) x previous-transition (common vs. rare) to be statistically significant ($\chi^2_{(1)}$=5.07, p=.02), but with a tiny effect-size (z-score=2.82, where the z-score describe the ratio between the unstandardized beta coefficient for the triple interaction and its standard-error). For comparison, we note that the paired interaction of transition x outcome yielded a z-score of 14.06, which is about five times bigger in size. Table S3 describe the descriptive estimates along with the difference in the outcome x transition paired interaction as a function of mapping repetition. We further analysed the outcome x transition pair interaction effect separately for each level of mapping (switch, repeat) and found a statistically significant effect for both trials with mapping-switches ($\chi^2_{(1)}$=182.91, p<.001, z-score=14.11) and mapping-repetitions ($\chi^2_{(1)}$=216.93, p<.001, z-score=15.41), suggesting that model-based decision making is apparent regardless of mapping. Therefore this should be considered a very small effect of mapping on model-based estimates, so that across participants, model-based estimated were slightly larger when the first-stage response-mapping repeated vs. switched compared to the previous trial.

Table S3. Stay probability in first-stage choices as a function of previous-transition, previous-reward and mapping-repetition

	Rewarded		Unrewarded		Interaction-score
	Common	Rare	Common	Rare	outcome x transition
Mapping repetition	80.16%	75.18%	68.49%	72.91%	9.4%
Mapping switch	75.21%	70.03%	64.24%	68.37%	9.3%

Next, we wanted to explore whether there is any relationship between reduced model-based scores due to response-mapping switches and a value spatial-motor associations effect at the first-stage. Specifically, we hypothesised that participants with greater spatial-motor model-free influences would have more difficulties representing the transition matrix due to mapping changes. This predicts that this increased influence of spatial-motor value associations should be positively related with a greater mapping effect on model-based estimates. To this end, we correlated the three model-agnostic scores (within state, between-states and between-stages) with the triple interaction of outcome x transition x mapping on

stay probability in the first-stage. We found no evidence for a relationship between the triple interaction and either within-state (r=.04, p=.28) and between-states effects (r=-.04, p=.24). We found a statistically significant effect for the between-stages effect (r=-.08, p=.03), opposite to the direction of the hypothesis tested.

In sum, we found a weak influence of spatial-motor mapping on model-based estimates. Our analysis was not consistent with an hypothesis that the transition matrix used by the model-based system was less accurate for participants with stronger influence of spatial-motor model-free associations.

Effect of reward on response-key sequences

Here, we examined whether response-key sequences made at both the first and second-stages get reinforced and carried over to the next trial. Following Dezfouli & Balleine (2013)(11), we hypothesized that if there is reinforcement of response-key sequences then the effect of reward on second-stage response-key selection will be greater when the first-stage response-key selection was repeated (i.e., indicating the influence of a response-key sequence). Alternatively, if there is no reinforcement of response-key sequences, we should expect no effect of first-stage response-key repetition on second-stage response-key reinforcement effects. We elaborate on the two effects indexing the influence of reward on response-key selection, within and between-states effect (see main text). Specifically, we consider whether these influences are stronger when the first-stage response-key selection is repeated in the *n*+1 compared with the *n* trial.

(a) Between-stage effect. We examined the stay probability with the same second-stage response-key in the *n+1* trial, as a function of previous-outcome (unrewarded vs. rewarded) and first-stage response-key repetition (switched, repeated). This was performed for trials where a different set of fractal was offered at the second-stage of *n* compared to *n+1* trials (i.e., between-states effect). We found a statistically significant outcome x first-key repetition effect ($\chi^2_{(1)}$=200.66, p<.001) such that the effect of reward on second-stage key repetitions was greater when the first-stage key selection was repeated vs. switched (see Figure S7). These results are strikingly similar to previous results reported using a two-step task where the motor action were action relevant (see Dezfouli & Balleine 2013 (11), Figure 5A), and suggest that first to second-stage response-key sequences were reinforced.

(b) Within-stage effect. We examined the probability of staying with the same second-stage fractal in the *n+1* trial, as a function of previous-outcome (unrewarded vs. rewarded), mapping-repetition (flipped vs. same) and first-stage response-key repetition (switched, repeated). This was done solely for trials where the same set of fractal was offered in the second-stage of the *n* compared to the *n*+1 trial (i.e., within-states effect). We found a statistically significant outcome x mapping-repetition x first-key repetition effect ($\chi^2_{(1)}$=20.06, p<.001) such that the paired interaction of outcome x mapping-repetition (indexing the influence of response-key reinforcement on choice, see main text) was greater when the first-stage response-key selection was repeated (see Figure S8).

We conclude that the effect of reward on second-stage response-key selection was stronger when the first-stage response-key selection was also repeated. This is in line with an hypothesis that response-key sequences were reinforced, despite being outcome-irrelevant.

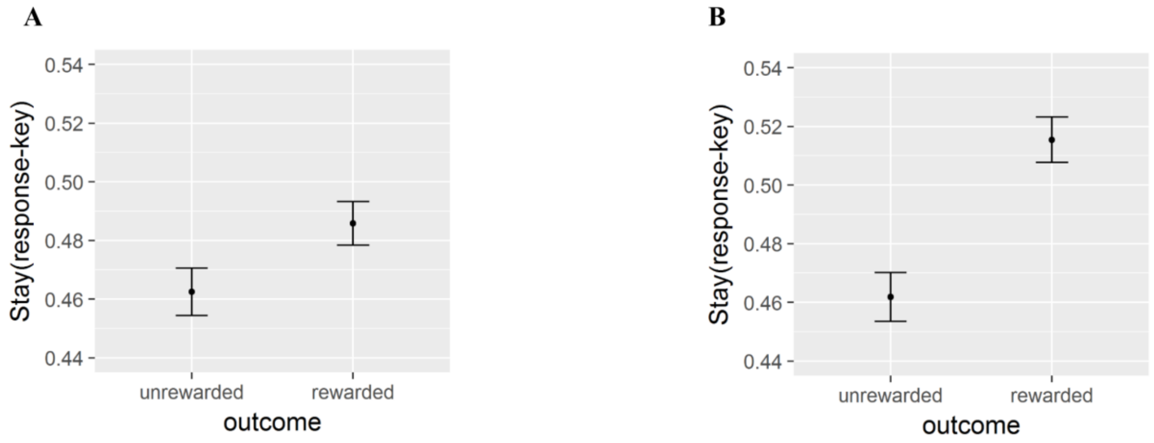

Figure S7. Response-key sequences. The figure presents an elaboration of the between-states sequential effect to also control for first-stage response-key repetitions. We demonstrate that when the first-stage response-key selection is repeated, reward show a greater influence on second-stage response-key stay probability. This effect suggest that in trials where a different set of fractals was offered in the *n*+1 vs. *n* trial, response-key sequences (first to second-stage) were reinforced and influenced choices in the *n*+1 trial.

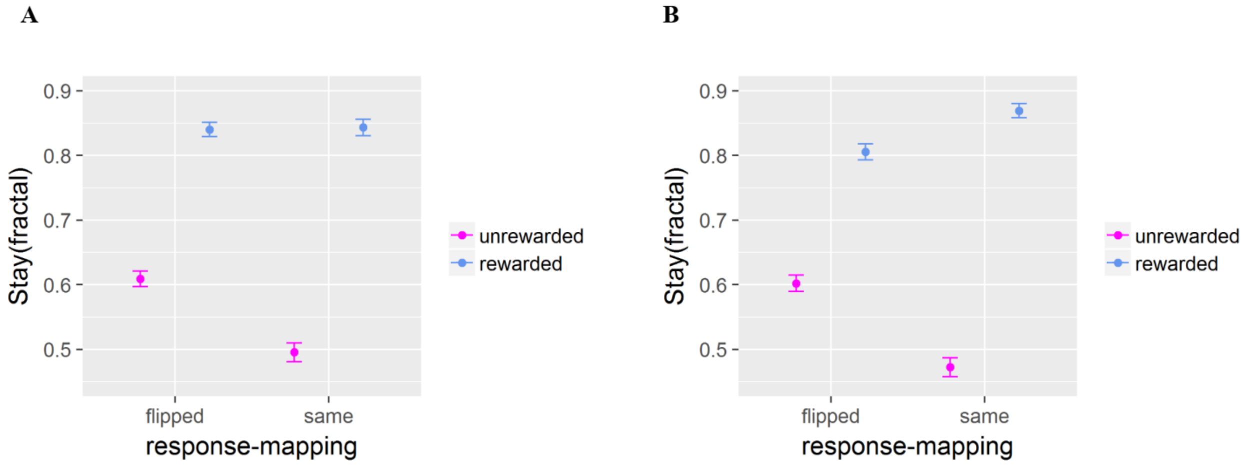

Figure S8. Response-key sequences when the same second-stage was repeated in *n* and *n+1* trials. The figure demonstrate the within-state effect (outcome x mapping-repletion interaction effect on fractal stay probability in the second stage) as a function of whether the first-stage response-key selection was switched (panel A) or repeated (panel B). This effect suggest that in trials where the same set of fractals was offered in the *n*+1 vs. *n* trial, response-key sequences (first to second-stage) were reinforced and influenced choices in the *n*+1 trial.

Examining the effects of random mapping repetitions on model-free spatial-motor associations

In the current task, fractal to response-key mapping was random, with each fractal equally likely to be associated with the right/left response-key each time it was offered. However, it might be that by chance, on some time windows the proportion of mapping repetitions was substantially higher, leading to a temporary relationship between the outcome-relevant fractals and spatial-motor aspect. Therefore, we tested whether a possible higher proportion of mapping-repetitions led to enhanced credit assignment to spatial-motor aspects of the task.

For this analysis, we divided the data into time windows (i.e., epochs) of 40 trials each. In each epoch we calculated the average proportion of mapping repetitions defined as:

$$P_{mapping-repeat}(\text{epoch}) = \left(\sum_{state=1}^{3} \frac{mapping_{repeat(state,epoch)}}{\text{visit}_{\text{repeat(state,epoch)}}}\right)/\ 3 \qquad \text{(S1)}$$

Where $\text{visit}_{\text{repeat}}$ is the number of trials the same state was visited in the *n* and *n+1* trials, and $\text{mapping}_{\text{repeat}}$ is the number of trials the same state appeared in the *n* and *n+1* trials with the same fractal to response-key mapping. We estimated also three model-agnostic scores estimating spatial-motor value associations for each of the corresponding epochs (i.e., within-state for first and second-stages, between-states and between-stages effects, see main text). We then performed three mixed effect regressions with $P_{\text{mapping-repeat}}$ predicting each of the model-agnostic scores estimating spatial-motor value associations for the corresponding epoch. Results (see Figure S9) showed no relationship with the within-state ($\chi^2_{(1)}$=0.26, p=.61) or between-states model-agnostic scores ($\chi^2_{(1)}$=1.2, p=.27). For the between-stages score we found a small effect in the opposite direction to our hypothesis ($\chi^2_{(1)}$=4.09, p=.02) so that on trials where the proportion of mapping repetitions was higher, there was a slight reduction on the between-stages effect (unstandardized slope of β=-.1). Overall these results provide no support for an hypothesis that a higher proportion of fractal to response-key mapping repetitions led to a stronger effect of spatial-motor value associations on behavior. Instead, it seems that value was assigned to spatial-motor aspects independently of those fluctuations.

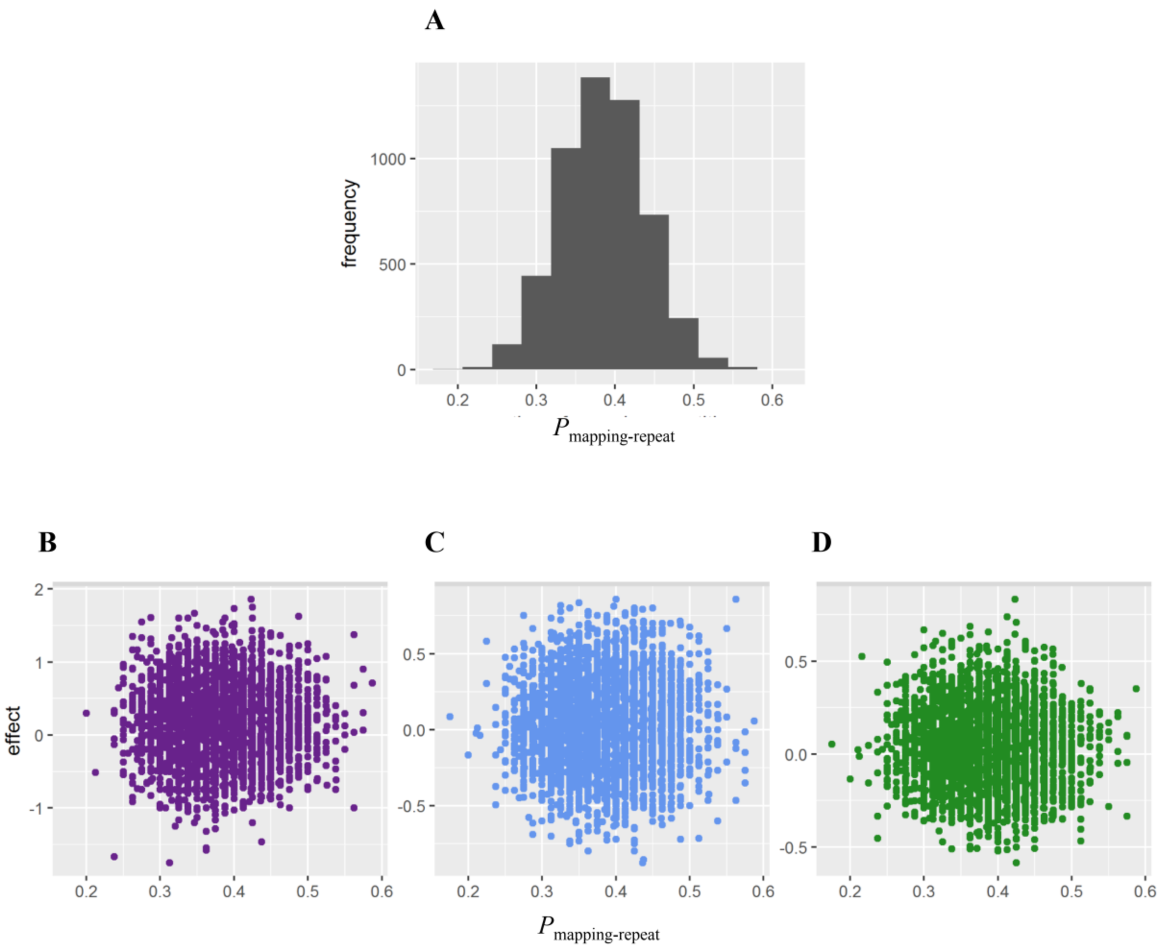

Figure S9. Effects of spatial-motor value associations as a function of random fractal to response-key repetitions. We divided the data into 40 trials time-windows and calculated the proportion of mapping repetitions ($P_{\text{mapping-repeat}}$; see Eq S1). (**A**) Histogram for the proportion of mapping-repetitions ($P_{\text{mapping-repeat}}$) across all time windows. (**B/C/D**) Scatters plots showing the relationship between the proportion of mapping repetitions ($P_{\text{mapping-repeat}}$; x-axis), the (**B**) within-state, (**C**) between-states (**D**) and between-stages effects for each time window. Results demonstrate high proportion of mapping repetition (due to chance) did not led to systematically higher spatial-motor value associations.

Spatial-motor effects in baseline measurement only

Our analyses were performed on a data-set that included up to three time measurements. Repeated measures might affect our main results such that, for example, on the second/third measurements participants might remember task structure and come with an already formed model-based strategy. For this reason we repeated the main analyses with Time-I data alone, where participants had no previous exposure to the task:

(a) We repeated the three model-agnostic sequential analysis, and found very similar results. This included a statistically significant effect for the within ($\chi^2_{(1)}$=216.18, p<.001) and, between states ($\chi^2_{(1)}$=68.26, p<.001) as well as between-stages ($\chi^2_{(1)}$=74.72, p<.001) effects.

(b) We repeated the model comparisons and found similar results to the full data set. Specifically, Model 3, 4 &5 performed worse compared to Model 1 where no spatial-motor learning took place (ΔBIC>=10933.32). Model 2 outperformed Model 1 (ΔBIC=231.22).

(c) We repeated the structural equation modeling analysis and found similar results to the one obtained with the full data-set, showing good model-fit [χ^2(13) 52.59, p<.001; RMSEA = .063 [.045 - .082]; CFI = .929; SRMR = .040] and a negative correlation between model-based and spatial-motor model-free latent factors (standardized covariance=-.22, p<.001).

References

1. Bates D, Maechler M, Bolker B, Walker S (2015) Fitting linear mixed-effects models using lme4. *Journal of Statistical Software* 67(1):1–48.

2. Daw ND, Gershman SJ, Seymour B, Dayan P, Dolan RJ (2011) Model-based influences on humans' choices and striatal prediction errors. *Neuron* 69(6):1204–1215.

3. Huys QJM, et al. (2011) Disentangling the roles of approach, activation and valence in instrumental and pavlovian responding. *PLoS Comput Biol* 7(4). doi:10.1371/journal.pcbi.1002028.

4. Gillan CM, Kosinski M, Whelan R, Phelps EA, Daw ND (2016) Characterizing a psychiatric symptom dimension related to deficits in goal-directed control. *eLife* 5. doi:10.7554/eLife.11305.

5. Decker JH, Otto AR, Daw ND, Hartley CA (2016) From creatures of habit to goal-directed learners: Tracking the developmental emergence of model-based reinforcement learning. *Psychol Sci* 27(6):848–858.

6. Deserno L, et al. (2015) Ventral striatal dopamine reflects behavioral and neural signatures of model-based control during sequential decision making. *Proc Natl Acad Sci U S A* 112(5):1595–1600.

7. Shahar N, et al. (2019) Improving the reliability of model-based decision-making estimates in the two-stage decision task with reaction-times and drift-diffusion modeling. *PLOS Comput Biol* 15(2):e1006803.

8. Rosseel Y (2012) lavaan: An R package for Structural Equation Modeling. *J Stat Softw* 48(1):1–36.

9. Schermelleh-Engel K, Moosbrugger H, Müller H (2003) Evaluating the fit of Structural Equation Models: Tests of significance and descriptive goodness-of-fit measures. *Methods Psychol Res* 8(2):23–74.

10. Kiddle B, et al. (2018) Cohort Profile: The NSPN 2400 Cohort: a developmental sample supporting the Wellcome Trust NeuroScience in Psychiatry Network. *Int J Epidemiol* 47(1):18-19g.

11. Dezfouli A, Balleine BW (2013) Actions, action sequences and habits: evidence that goal-directed and habitual action control are hierarchically organized. *PLoS Comput Biol* 9(12):e1003364.

Neuroscience in Psychiatry Network (NSPN) Consortium author list

Principal investigators:
Edward Bullmore (CI from 01/01/2017)
Raymond Dolan
Ian Goodyer (CI until 01/01/2017)
Peter Fonagy
Peter Jones

NSPN (funded) staff:
Michael Moutoussis
Tobias Hauser
Sharon Neufeld
Rafael Romero-Garcia
Michelle St Clair
Petra Vértes
Kirstie Whitaker
Becky Inkster
Gita Prabhu
Cinly Ooi
Umar Toseeb
Barry Widmer
Junaid Bhatti
Laura Villis
Ayesha Alrumaithi
Sarah Birt
Aislinn Bowler
Kalia Cleridou
Hina Dadabhoy
Emma Davies
Ashlyn Firkins
Sian Granville
Elizabeth Harding
Alexandra Hopkins
Daniel Isaacs
Janchai King
Danae Kokorikou
Christina Maurice
Cleo McIntosh
Jessica Memarzia
Harriet Mills
Ciara O'Donnell
Sara Pantaleone
Jenny Scott

Affiliated scientists:
Pasco Fearon
John Suckling

Anne-Laura van Harmelen
Rogier Kievit
